# Diphtheria

**Published:** 1874-03

**Authors:** J. J. Grace

**Affiliations:** Alabama


### DIPHTHERIA.

BY J. J. GRACE, M.D., OF ALABAMA.

Seeing that diphtheria has been, and is still making its ravages through the country, and that many physicians do not understand its treatment, I will give you my experience with it for the last three years, having never lost a case in my practice. I apply turpentine and flannel cloths to the throat; use nit. silver, from 10 to 30 grs. to ounce of rose-water, with camel's hair pencil, to tonsils and glands, every 24 or 36 hours; and give the following mixture, viz: ℞. Quinine sulph. gr. xvi.; chlorate potass., ʒi.; tinc. ferri chlo. ʒii.; syr. simple, ʒiv.; aqua g., ℥ii. M. A teaspoonful every three hours, and keep the bowels open with magnesia or castor oil every day. With children from one to four years, this is an unfailing remedy, and older persons should have the dose increased.

